# Alfalfa (*Medicago sativa* L.)/Maize (*Zea mays* L.) Intercropping Provides a Feasible Way to Improve Yield and Economic Incomes in Farming and Pastoral Areas of Northeast China

**DOI:** 10.1371/journal.pone.0110556

**Published:** 2014-10-16

**Authors:** Baoru Sun, Yi Peng, Hongyu Yang, Zhijian Li, Yingzhi Gao, Chao Wang, Yuli Yan, Yanmei Liu

**Affiliations:** Key Laboratory of Vegetation Ecology, Northeast Normal University, Changchun, China; Agroecological Institute, China

## Abstract

Given the growing challenges to food and eco-environmental security as well as sustainable development of animal husbandry in the farming and pastoral areas of northeast China, it is crucial to identify advantageous intercropping modes and some constraints limiting its popularization. In order to assess the performance of various intercropping modes of maize and alfalfa, a field experiment was conducted in a completely randomized block design with five treatments: maize monoculture in even rows, maize monoculture in alternating wide and narrow rows, alfalfa monoculture, maize intercropped with one row of alfalfa in wide rows and maize intercropped with two rows of alfalfa in wide rows. Results demonstrate that maize monoculture in alternating wide and narrow rows performed best for light transmission, grain yield and output value, compared to in even rows. When intercropped, maize intercropped with one row of alfalfa in wide rows was identified as the optimal strategy and the largely complementary ecological niches of alfalfa and maize were shown to account for the intercropping advantages, optimizing resource utilization and improving yield and economic incomes. These findings suggest that alfalfa/maize intercropping has obvious advantages over monoculture and is applicable to the farming and pastoral areas of northeast China.

## Introduction

The farming and pastoral area (FPA) of northeast China (NEC) is an agriculture-based ecozone combining forestry and animal husbandry, and it is an important grain commodity and animal husbandry base. However, it is also a vulnerable eco-environmental zone owing to low vegetation cover, fine sandy soil and strong wind [1]–[2]. Wind erosion, water erosion and unsustainable production activities (e.g., single cropping and multiple-year continuous cropping) have made the land subject to dust storms in winter and spring. Moreover, cropland soil is being increasingly eroded, causing low soil fertility and reduced crop productivity and quality [3]–[5]. Together with the growing use of agricultural chemicals, such as fertilizers and herbicides, sustainable agricultural development is now facing many serious challenges [6].

In addition to the cropland, grassland has been degraded due to overgrazing and excessive agricultural reclamation, and grassland degradation is reflected by the reduced grassland production and forb quality and low carrying capacity [7]. “Grain for green” initiatives including reseeding of forage grasses have long been considered as effective ways to restore grassland vegetation and help to balance the ecological system [8]. Moreover, with increasing demand for meat products and high quality forage grass, China continues to give substantial support and increased financial investment to the development of the forage industry. Additionally, the Chinese government has strongly endorsed a proposal for boosting the development of the alfalfa industry so as to ensure the production, processing and sustainable supply of high quality forage grass [9]. Therefore, in the context of food and eco-environmental security and animal husbandry sustainable development, traditional farming patterns in the northeast FPA are being altered; with a tendency to adjust agricultural structure, introduce forage grass into the main crop farming system and establish an intercropping pattern between crops and forage grass, resulting in efficient resource utilization, a friendly ecological environment and good economic benefits [10]–[11].

Intercropping, the practice of growing two or more crops in proximity, is advantageous due to the differences in ecological characteristics and growth of the intercropped varieties. This can establish a composite population, producing complementary effects and increasing yield and economic incomes per unit area [12]. Furthermore, intercropping can improve soil fertility, alleviate disease and insect harm, and inhibit the growth of weeds [13]–[16]. Maize, as a principal crop of the northeast FPA, is an important food and forage crop. Its grain is an important fodder with high energy, known as “queen feed” [17]. Alfalfa is a leguminous forb that has been prioritized in the development of the PFA of NEC; suitable because of its high yield, rich content and high quality of protein, abundant vitamins and minerals, good palatability and high digestibility [18]. Moreover, as a perennial, alfalfa supplies soil cover throughout the year; providing wind resistance, fixing soil and improving the environment of the planting area. Its large root system can significantly improve soil fertility and physico-chemical properties, leading to the win-win relationship between utilization and conservation [19]. Therefore, it is predictable that intercropping alfalfa with maize can not only guarantee regional food security and meet the nutritional requirements of forage industry, but also provide eco-environmental protections, and it is a promising cropping pattern in the future development of this region.

In China, maize has been traditionally cultivated in even rows. More recently, in attempts to improve maize yield agricultural scientists have experimented with alternative cultivation strategies. Indeed, a study has shown that in the main agricultural region of Jilin Province, planting maize in alternating wide and narrow rows can achieve up to 10% improved yield over the conventional pattern [20]. Previous intercropping studies focused mainly on food crop combinations [21]–[22], but few studies have investigated the possibility of intercropping alfalfa with maize, a strategy combining annual food crop with perennial forage crop together. Those studies that have addressed alfalfa/maize intercropping focused on improving group yield, forage quality, soil fertility and the environment [23]–[24]. To our knowledge, no study has investigated whether intercropping alfalfa with maize in alternating wide and narrow rows could provide sustained high yield and economic incomes while taking investment and environmental factors into account, and whether there are some constraints limiting its popularization such as crop management and the acceptability of local farmers. Hence, a field experiment was conducted to explore the following questions: (1) Compared to an even row approach, does an alternating wide and narrow row planting approach in maize improve yield in the FPA of NEC? (2) Can intercropping alfalfa with maize provide sustained high yield and economic incomes? Which intercropping mode is best, considering farmer incomes and land management?

## Materials and Methods

### Experimental site

**Figure 1:**
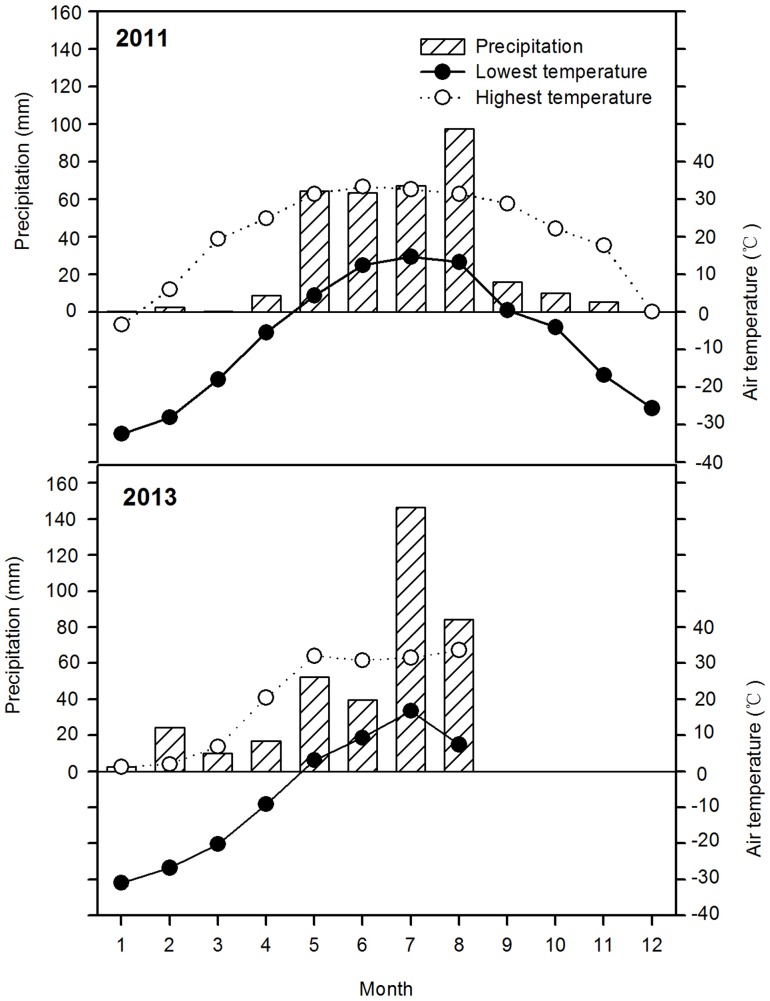
Monthly precipitation (bar) and air temperature (curve) of the experimental site in 2011 and 2013.

The study was conducted between 2007 and 2013 at the Grassland Ecosystem Field Station of the Northeast Normal University at Songnen Grassland (123° 44′ E and 44° 40′ N, 137.8–144.8 masl), a typical FPA of NEC. This area is characterized by a semi-arid and temperate continental monsoon climate with a mean annual temperature of 4.6–6.4°C, an annual accumulated temperature (≥10°C) of 2546–3375°C, mean annual precipitation of 300–400 mm (86% of precipitation occurring from May to September) and mean annual evaporation of 1500–2000 mm. The frost-free period lasts approximately 140 days, from the end of April to early October. The two experimental years contrasted each other in terms of precipitation. In 2011, the annual total precipitation was 335 mm and mostly occurred in the growing season (308 mm), whereas the year 2013 was a year with a higher precipitation amount (376 mm from January to August), better seasonal distribution and pronounced peak in July. Air temperature in 2011 and 2013 showed a similar dynamic with the maximum and minimum air temperatures of 33°C and −32°C, respectively (Figure 1). The soil type at the site is light chernozem with deep soil layers. The plough layer consists of organic C (17.24±1.76 g kg^−1^), total N (0.98±0.15 g kg^−1^), rapidly available P (5.88±0.65 mg kg^−1^) and rapidly available K (140.70±11.75mg kg^−1^), with an initial soil pH of 7.46±0.04.

### Experiment materials and design

Alfalfa variety *Medicago sativa* L. cv. Dongmu No. 1 was used throughout this study. This variety was bred by Northeast Normal University to adapt readily to drought and cold and is now the principally cultivated variety in the study area. Alfalfa generally turns green in mid-April, continues to grow until end of October and can be harvested three times per year. The hay yield can reach up to 7500–10,000 kg ha^−1^ in the second year under the rainfed condition. *Zea mays* L. cv. Zhengdan 958 was chosen as the maize test variety. This variety matures at about 128 days and is widely cultivated by local farmers due to the high and stable yield and a great resistance to lodging and disease. The differences of alfalfa and maize in growth dynamics (Figure S4) make them easy to form temporal and spatial complementarity and promote the efficient utilization of light, water and nutrients.

At the beginning of the experiment, the whole field was fully ploughed to ensure uniform soil conditions. The experiment was conducted in a completely randomized block design with four blocks each containing five cropping patterns: maize monoculture in even (65 cm) rows (MME); maize monoculture in alternating wide (90 cm) and narrow rows (40 cm) (MMW); alfalfa monoculture in even (30 cm) rows (MA); MMW intercropped with alfalfa, with one row of alfalfa in the wide rows (23.1% alfalfa in intercropping area) (IMA1); MMW intercropped with alfalfa, with two rows of alfalfa in the wide rows (46.2% alfalfa in intercropping area) (IMA2). In the maize planting patterns, every three rows of maize were defined as one belt (1.3 m wide) with three belts in each treatment. Each plot had an area of 46.8 m^2^ (3.9 m × 12 m), with 50 cm spacing between each plot and 1 m separating each block.

To establish the intercropping system, alfalfa was sown in early July 2007 at a seeding rate of 15 kg ha^−1^ and had been allowed to grow for 4 years before data collection in 2011. Every year maize was sown in early May with 26 cm separating each plant, and was irrigated with 75 mm before its sowing to ensure good germination. In all planting patterns, 135 kg P ha^−1^ and 90 kg K ha^−1^ were applied for alfalfa, and 225 kg N ha^−1^, 120 kg P ha^−1^ and 60 kg K ha^−1^ were applied for maize. The commercial fertilizer used were: nitrogen, urea (46% nitrogen content); phosphate, diammonium phosphate (46% phosphorus content, 18% nitrogen content); potash, potassium chloride (60% potassium content). All fertilizers required by alfalfa were spread in the soil at the time of sowing. For maize, all of the phosphate and potash, and half of the nitrogen fertilizers were spread in the soil at the time of sowing and the remaining nitrogen fertilizers applied during the big flare opening period. Weeds were regularly controlled using a hand hoe, and pest and disease of alfalfa or maize were separately controlled timely with the idea of minimizing the pesticide application effects on the non-target crop.

### Data collection

The data was collected from 2011. Unfortunately, there was considerably small snowfall and the air temperature was relatively high in the winter of 2011 (Figure 1), which weakened alfalfa resistance to cold and freezing [25]. In the early spring of 2012, the sprout of alfalfa was promoted due to the continuously high air temperature from 21^th^ to 30^th^ in March with the maximum 16.8°C, whereas the air temperature dramatically decreased from 31^th^ March to 6^th^ in April with the minimum −9.0°C (Figure S1). This unexpected cold snap made a serious freezing injury to the sprouting alfalfa due to its lowest cold resistence at that time [26]. Consequently, alfalfa achieved a low turning green rate. In order to ensure the sustainable production of alfalfa, we stopped data collection in 2012 and restarted sampling in 2013 when alfalfa turned a good recovery in its growth and development.

Light intensity was determined using a ST-80C illuminometer (Photoelectric Instrument Factory of Beijing Normal University, China) in 2011. Four layers for maize or alfalfa were selected in each treatment: (1) the reference layer, above the canopy; (2) the bottom layer, close to the soil surface; (3) the intermediate layer, at the point of 1/2 plant height and (4) the top layer, 10 cm below the top of the plant. Based on the light intensity, light transmission was calculated for each of the layers. Maize and alfalfa leaf area index (LAI) was measured using a LAI-2000 plant canopy analyzer (LI-COR, Inc., USA) in 2011 and 2013. From the first flowering stage of alfalfa, both light intensity and LAI were tested from 10:00–11:00 on a sunny day. The measurement was repeated every 15 days at three different positions within each testing belt.

Using time domain reflectometry (TDR 100, Campbell Scientific Inc., Logan, Utah, USA), soil water content (SWC) at depth of 0–20 cm was measured four times for all the treatments in each year (2011: 10^th^ June, 24^th^ July, 10^th^ September and 4^th^ October; 2013: 6^th^ June, 12^th^ July, 26^th^ August and 30^th^ September). The measuring time was corresponding to different developing stages of crop, that was the first, second and third flowering stage of alfalfa and the maturity stage of maize, respectively. Specifically, SWC was tested at ten different representative positions between the alfalfa or maize rows in the monoculture treatments for each plot. As to intercropping treatments, SWC was measured between the alfalfa rows (for IMA2), between the alfalfa and maize rows and between the maize rows with ten different representative positions in each belt of plots and then all measured data were averaged as the SWC condition of the intercropping system.

The final harvest of maize was taken in early October (2011: 5^th^ October; 2013: 1^th^ October). In each planting pattern, the second belt was selected for grain yield determination. Fresh weight was recorded before maize grains were oven-dried at 65°C to a constant weight and dry weight recorded. Water content and grain yield were calculated based on dry weight. Alfalfa was cut three times (2011: 11^th^ June, 25^th^ July and 11^th^ September; 2013: 7^th^ June, 13^th^ July and 27^th^ August). For each alfalfa cut, fresh weight and dry weight were determined, and water content and hay yield calculated.

### Data calculations

#### Light transmission

Light transmission (LT) was calculated using equation (1);
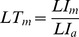
(1)


where m is the canopy layer (including the top, intermediate and bottom layers) of either alfalfa or maize, LT_m_ is the light transmission at layer m, LI_m_ is the light intensity at layer m, and LI_a_ is the light intensity above the canopy [27].

#### Output value per unit area

The output value per unit area (OVPUA) was calculated according to equation (2);
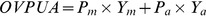
(2)


where for each planting pattern, P_m_ and P_a_ denote the price of maize grain and alfalfa hay respectively. The price expressed in USD is based on the exchange rate of 630 ¥ 100 USD^−1^ in 2011 and 613 ¥ 100 USD^−1^ in 2013, thus P_m_ and P_a_ are respectively 361.90 USD t^−1^ and 380.95 USD t^−1^ in 2011 and 342.58 USD t^−1^ and 358.89 USD t^−1^ in 2013 based on local market values; Y_m_ and Y_a_ denote the yield of maize grain and alfalfa hay respectively [28].

#### Land equivalent ratio

Land equivalent ratio (LER) is an index that adopts yield as a comparison parameter to evaluate land use efficiency of different cultivated patterns relative to monoculture. However, the value of LER is not necessarily related to yield. The equation is defined as follows;
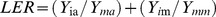
(3)


where Y_ia_ and Y_im_ are the respective yields of alfalfa and maize in the total intercropped area, and Y_ma_ and Y_mm_ are the yields of monocultured alfalfa and maize. An LER greater than 1.0 reveals an intercropping advantage and the favors of intercropping on crops growth and yield, while an LER less than 1.0 indicates an intercropping disadvantage and the negative affections of intercropping on crops growth and yield [29]–[31].

#### Aggressivity

Aggressivity (A_ac_) measures the relative resource competitiveness of two intercropped species; 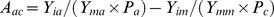
(4)


where A_ac_ is the aggressivity of alfalfa relative to maize in the intercropping system, P_a_ and P_c_ are the intercropping area proportions occupied by alfalfa and maize respectively, while the meanings of other symbols are the same as those used for the LER equation. If A_ac_ is greater than 0, the competitive ability of alfalfa exceeds that of maize in intercropping; otherwise, maize has greater competitiveness [12], [23].

### Statistical analysis

Normal distribution and homogeneous variances were tested for all the data with Shapiro-Wilk test [32] using SPSS 17.0 software (SPSS Inc., Chicago, IL, USA), and light transmission was biquadrate after reciprocal transformed to achieve normal distribution. One-way ANOVA was performed to examine the effects of cropping patterns on light transmission and soil water content (SWC). Repeated measures ANOVA in a general linear model (GLM) were conducted to assess the effects of planting modes on LAI, yield and output value per unit area (OVPUA), with year and alfalfa flowering stage as the repeated measures. The results were reported using the Greenhouse-Geisser correction when Mauchly's test of sphericity was violated. If the interaction between factors was significant, one-way ANOVA was conducted to evaluate the effects of cropping pattern or alfalfa flowering stage and significant differences of means were compared with Duncan's multiple-compare range test; while the effects of year were tested by independent-samples t test. Significant level was set at *P* <0.05.

## Results

### Maize light transmission

**Figure 2:**
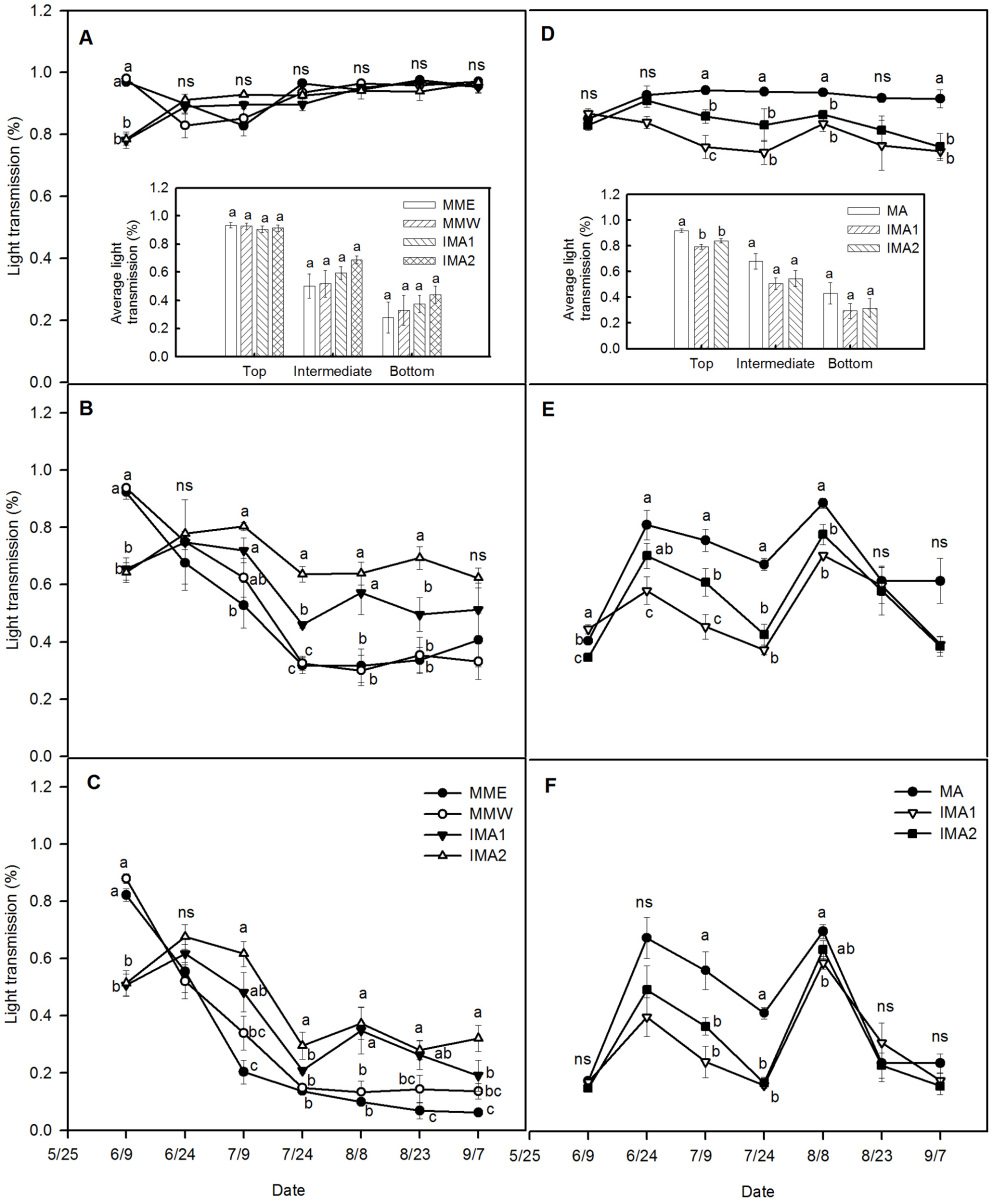
Light transmission dynamics of maize and alfalfa at different layers under monoculture and intercropping. The inset figures show the average light transmission of maize and alfalfa in different planting patterns during the vegetation period. A  =  top light transmission of maize, B  =  intermediate light transmission of maize, C  =  bottom light transmission of maize, D  =  top light transmission of alfalfa, E  =  intermediate light transmission of alfalfa, F  =  bottom light transmission of alfalfa. MME  =  monoculture maize in even rows, MMW  =  monoculture maize in alternating wide and narrow rows, MA  =  alfalfa monoculture, IMA1  =  maize intercropped with one row of alfalfa in the wide rows, IMA2  =  maize intercropped with two rows of alfalfa in the wide rows. Different letters for the same date indicate significant difference at *P* <0.05 probability level, and ns represents no difference between treatments. Values  =  means ± SE.

With the exception of the first flowering stage of alfalfa (early June), no significant difference was found in light transmission at the top of maize among treatments (Figure 2A). At the stage of the first flowering of alfalfa, light transmission of monoculture maize (MME and MMW) was significantly higher than that of intercropped maize (IMA1 and IMA2) (*P* <0.0001), whereas there was no significant difference between monoculture modes or between intercropping modes. This can be accounted for by the fact that during flowering, alfalfa grew taller than maize, which had an overshadowing effect. On the whole, however, no significant difference was observed for average light transmission at the top of maize, regardless of cropping pattern (Figure 2A inset).

During continuous growth of maize, there was a tendency for a reduction in both intermediate and bottom light transmission for all treatments. This was particularly evident before maize entered the big flare opening stage (24^th^ July), with little change in light transmission occurring after this point (Figure 2B and C). In the first flowering stage of alfalfa, both intermediate and bottom light transmission of monoculture maize (MME and MMW) were higher than for intercropped maize (IMA1 and IMA2); however, the opposite pattern was observed in the resting period, when light transmission of IMA1 and IMA2 maize was higher than monoculture maize (Figure 2B and C). In addition, there were significant differences for intermediate and bottom light transmission between intercropping and monoculture maize at the second alfalfa flowering stage (24^th^ July) (intermediate: *P* <0.0001; bottom: *P* = 0.003) and after the second cutting of alfalfa (8^th^ August) (intermediate: *P* = 0.002; bottom: *P* = 0.005). Significant differences were also found for intermediate and bottom light transmission between intercropped maize (IMA1 and IMA2) at the second flowering stage of alfalfa, but differences between monocultures of maize were never significant. However, for maize intermediate or bottom canopy layers, no significant difference in average light transmission was observed, regardless of planting strategy (Figure 2A inset).

### Alfalfa light transmission

Throughout the alfalfa growing season, there was no evident variance in top light transmission in any treatment, whereas the intermediate and bottom light transmission dynamics of all treatments showed a bimodal curve (Figure 2D, E and F), which can be attributed to alfalfa being cut twice (11^th^ June and 25^th^ July). Before cutting, alfalfa was flowering and therefore the canopy had high closure and lower light transmission. Upon cutting, light transmission increased. Subsequent alfalfa regrowth produced new canopy closure and decreased light transmission.

With the exception of the first, second and sixth measuring times, the top layer light transmission of monoculture alfalfa (MA) was significantly higher than that of intercropped alfalfa at all recorded time points (third: *P* = 0.003; forth: *P* <0.0001; fifth: *P* = 0.004; seventh: *P* = 0.010). The season average light transmission of MA was significantly higher than that of intercropped alfalfa. There was no significant difference between the two intercropping modes (Figure 2D and inset). In the intermediate and bottom layers, light transmission showed complex patterns with time. In the intermediate layer, light transmission of MA was significantly higher than in the two intercropping models at the third, fourth and fifth measuring times (third: *P* = 0.003; forth: *P* <0.0001; fifth: *P* = 0.003). With the exception for the first and third measuring times, there was no significant difference between the light transmissions of the two intercropping modes (Figure 2E). In the bottom layer, for the third and fourth measuring times, light transmission of MA was significantly higher than the two intercropping modes (third: *P* = 0.006; forth: *P* <0.0001) and light transmission of the two intercropping modes showed no significant difference (Figure 2F). For intermediate and bottom layers, there were no significant differences in the season average light transmission among treatments (Figure 2D inset).

### Soil water content (SWC)

**Figure 3:**
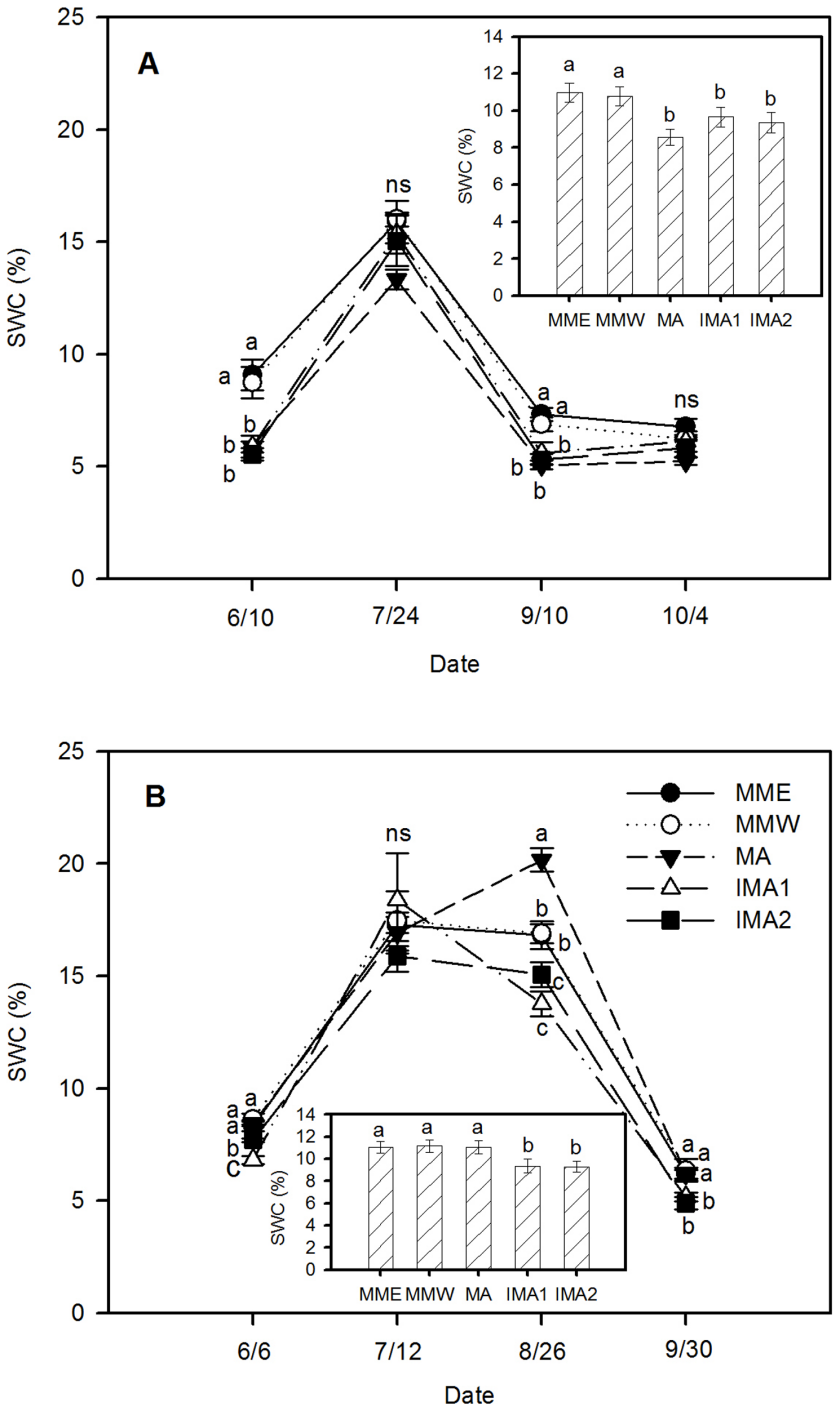
Soil water content comparisons of different cropping patterns in 2011 (A) and 2013 (B). The inset figures show the average soil water content in different planting patterns during the vegetation period. SWC  =  soil water content. The other symbols are the same as for Figure 2.

SWC of all cropping patterns displayed a strong seasonal dynamic, with a peak in July and August (Figure 3). Irrespective of the growing stage, the difference of SWC between MME and MMW was not significant in both 2011 and 2013. Compared to monoculture, intercropping significantly reduced the SWC, and it was more evident in 2013 than 2011 (Figure 3). In 2011, with the exception of 24^th^ July and 4^th^ October, the SWC of IMA1 and IMA2 was significantly decreased compared to MMW, but with no significant difference compared with MA (both: *P* <0.0001) (Figure 3A); while in 2013, the SWC of IMA1 and IMA2 was significantly lower than that of MMW as well as MA except for 12^th^ July (all: *P* <0.0001) (Figure 3B). The differences between treatments were also reflected by seasonal average SWC: the values of IMA1 and IMA2 were significantly lower than that of MMW and MME in both 2011 and 2013, while there was no significant difference between IMA1 and IMA2 in both years. For the MA treatment, the seasonal average SWC was significantly lower than that of MMW and MME in 2011, while there was no significant difference among treatments MMW, MME and MA in 2013 (Figure 3 3A inset and 3B inset).

### Leaf area index (LAI)

**Figure 4:**
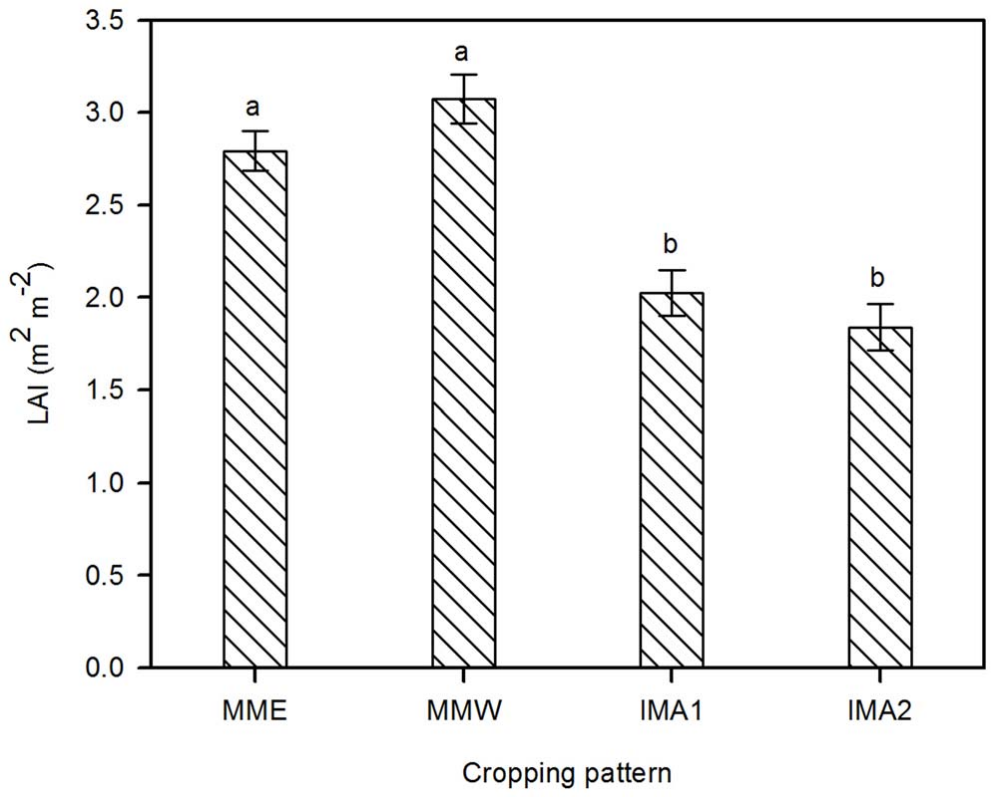
Leaf area index comparisons of maize at the harvest stage under monoculture and intercropping. Significant differences between different cropping patterns are indicated by lower case letters (*P* <0.05). The other symbols are the same as for Figure 2.

**Figure 5:**
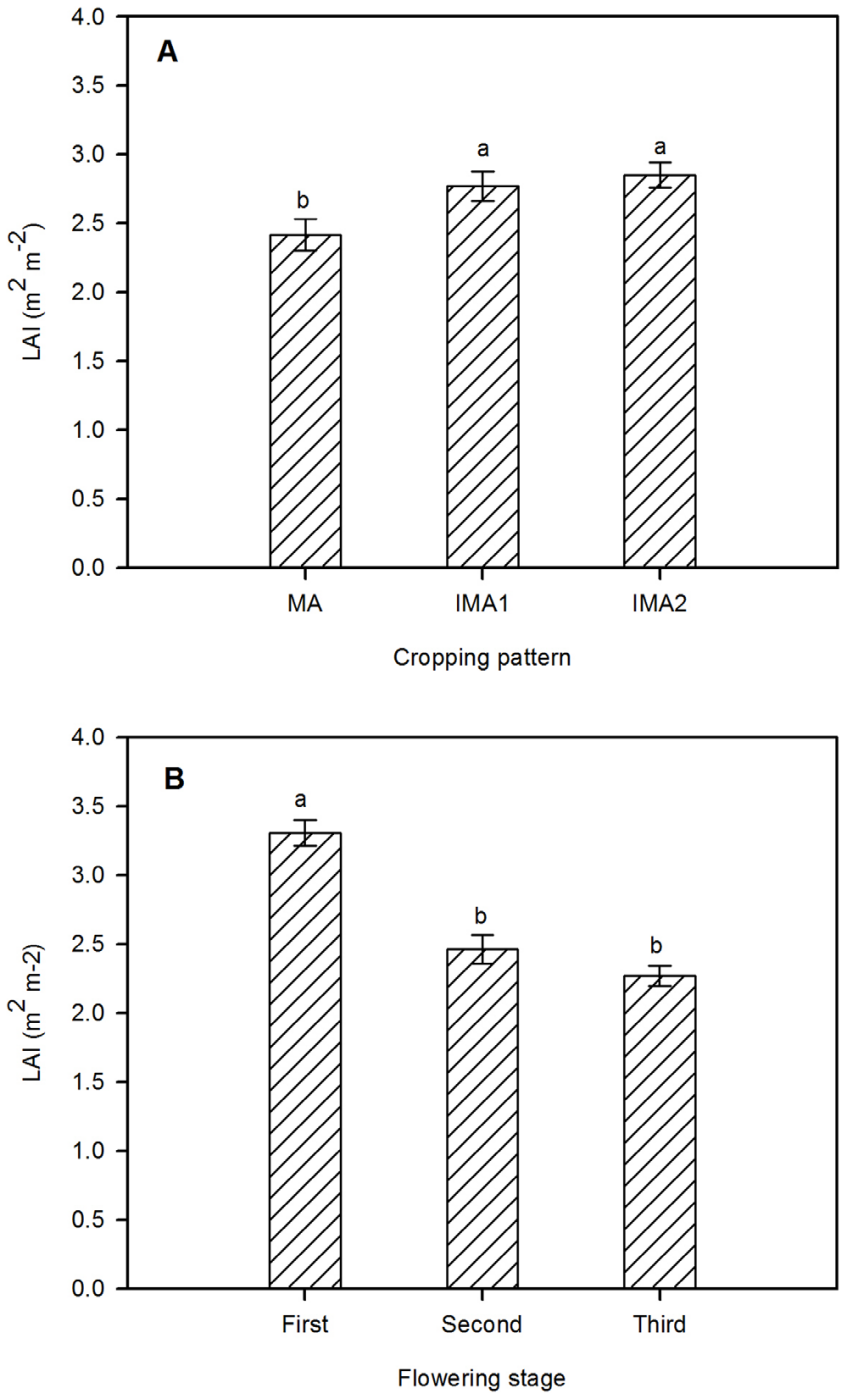
Leaf area index comparisons of alfalfa under monoculture and intercropping (A) and at different flowering stages (B). Significant differences between different alfalfa flowering stages are indicated by lower case letters (*P* <0.05). The other symbols are the same as for Figure 2 and 4.

There was no significant difference in maize LAI between MME and MMW (Figure 4). Compared to MMW, the LAI of intercropped maize (IMA1 and IMA2) was significantly reduced (*P* <0.0001), and no significant difference was observed between the two intercropping patterns (Table 1; Figure 4). Regarding alfalfa LAI, the values of IMA1 and IMA2 were significantly higher than that of MA (*P* = 0.009), but with no significant difference between IMA1 and IMA2 (Table 1; Figure 5A). Meanwhile, alfalfa LAI was significantly affected by its flowering stage (*F* = 32.648, *P* <0.0001), LAI in the first flowering stage was significantly higher than that in the second and third flowering stages (*P* <0.0001) (Figure 5B).

**Table 1:** Results of repeated measures ANOVA on maize leaf area index (LAI) and yield, alfalfa yield and comprehensive benefit analysis of total yield and output value per unit area (OVPUA), with cropping pattern (CP) as the independent variable and year (Y) as the repeated measure.

Factors	Maize			Alfalfa		Comprehensive benefit analysis		
	Df	LAI	Yield	Df	Yield	Df	Total yield	OVPUA
CP	3	26.691*	49.157**	2	430.879**	4	16.343**	15.063**
Y	1	0.349 ns	21.174**	1	0.248 ns	1	32.515**	8.052*
CP × Y	3	0.846 ns	9.024**	2	3.985 ns	4	10.850**	10.699**

Df  =  degrees of freedom, ns  =  no significant difference, * *p* <0.05, ** *p* <0.01

### Yield and output value per unit area (OVPUA)

Although both grain yield and OVPUA of MMW were higher (6.8% in 2011 and 6.5% in 2013) than the corresponding values of MME, the difference between the two cultivated patterns was not statistically significant (Table 2). Compared to monoculture, alfalfa hay yield in the intercropping treatments increased significantly, while maize grain yield in the same treatments was reduced dramatically in both years (all: *P* <0.0001). The corresponding parameters in the IMA1 cropping pattern were altered in a greater extent than that in IMA2. Additionally, maize grain yield of IMA1 in 2013 was significantly higher than that in 2011 (*P* = 0.028) (Table 1 and 2).

**Table 2:** Comparisons of total yield and OVPUA of different planting patterns.

Treatment	Yield (t ha^−1^)			OVPUA (USD ha^−1^)			LER	A_ac_
	Maize	Alfalfa	Total	Maize	Alfalfa	Total		
**2011**								
MME	11.00±0.33a	–	11.00±0.33a	3982.31±120.85	–	3982.31±120.85a	–	–
MMW	11.75±0.33a	–	11.75±0.33a	4253.31±120.38	–	4253.31±120.38a	–	–
MA	–	7.85±0.31a	7.85±0.31b	–	2991.92±118.56	2991.92±118.56b	–	–
IMA1	5.87±0.34 (7.63±0.44b)	6.08±0.53 (26.35±2.29b)	11.95 ± 0.30a	2124.17±122.92	2316.91±201.23	4441.08±116.95a	1.27	2.70
IMA2	3.71±0.64 (6.89 ± 1.19b)	7.21±0.34 (15.63±0.74c)	10.92±0.67a	1342.17±231.66	2747.93±129.75	4090.10±246.77a	1.23	1.40
**2013**								
MME	10.68±0.29a	–	10.68±0.29a	3656.77±100.28	–	3656.77±100.28a	–	–
MMW	11.37±0.23a	–	11.37±0.23ab	3894.69±79.08	–	3894.69±79.08a	–	–
MA	–	11.76±0.05a	11.76 ± 0.05b*	–	4221.72±18.90	4221.72±18.90b*	–	–
IMA1	7.18±0.18 (9.34±0.23b*)	5.79±0.27 (25.06±1.17b)	12.97±0.19c*	2460.38 ± 61.59	2075.89±97.10	4536.26±67.64c	1.12	1.31
IMA2	4.77±0.15 (8.87 ± 0.27b)	7.79±0.11 (16.88±0.23c)	12.56 ± 0.05c	1633.92±50.89	2795.73±38.22	4429.65±14.12c	1.08	0.65

MME  =  monoculture maize in even rows, MMW  =  monoculture maize in alternating wide and narrow rows, MA  =  alfalfa monoculture, IMA1  =  maize intercropped with one row of alfalfa in the wide rows, IMA2  =  maize intercropped with two rows of alfalfa in the wide rows. Values in the parentheses are yields based on the whole of the intercropping area, including the areas occupied by both maize and alfalfa, and are equal to the yields of maize or alfalfa divided by their respective area proportion. The intercropping area proportions of maize and alfalfa were respectively 76.9% and 23.1% in the IMA1 treatment, while the intercropping area ratios occupied by alfalfa and maize were 53.8% and 46.2% in the IMA2 treatment. Different letters in the same column following the values indicate significant difference between different cropping patterns, and * denotes significant difference between years (*P* <0.05). Value  =  mean ± S.

The comprehensive benefits for total yield and OVPUA were significantly affected by the interaction between cropping pattern and year (Table 1). In 2011, both total yield and OVPUA of IMA1 and IMA2 were significantly enhanced compared to MA (both: *P* <0.0001), while no significant increase was found compared to MMW. In 2013, both total yield and OVPUA of the two intercropping patterns were significantly higher than that of MA as well as MMW (both: *P* <0.0001). In terms of year effects, both total yield and OVPUA of MA in 2013 was significantly higher than that in 2011 (total yield: *P* = 0.038, OVPUA: *P* <0.0001), and there was also a significant increase in the total yield of IMA1 in 2013, as compared to 2011 (*P* <0.0001) (Table 2).

Furthermore, land equivalent ratios (LERs) of IMA1 and IMA2 were 1.27 and 1.23 in 2011 and 1.12 and 1.08 in 2013, respectively, demonstrating that both intercropping strategies were advantageous, and that the IMA1 pattern was superior to IMA2. The calculated aggressivity (A_ac_) values for IMA1 and IMA2 were 2.71 and 1.40 in 2011 and 1.31 and 0.65 in 2013, respectively, demonstrating that the resource competitiveness of alfalfa was greater than that of maize in the two intercropping systems (Table 2).

## Discussion

### Maize monoculture in wide and narrow rows vs. in even rows

Liu et al. [20] reported that in the main agricultural region of Jilin Province, compared to even rows, maize planted in alternating wide and narrow rows increased group light transmission, photosynthetic potential, leaf area and grain yield by more than 10%. The results presented here are in agreement with these findings; both grain yield and output value of MMW were enhanced by 6.8% in 2011 and 6.5% in 2013 relative to MME (Table 2). This was mainly attributable to the improved spatial structure of the group, which increased light transmission (Figure 2), improved maize growth conditions and promoted the formation of edge effect [33]. At harvest time, maize achieved a greater LAI and dry matter accumulation (Figure 4 and S3). Therefore, in the PFA of NEC, maize cropped in alternating wide and narrow rows also had a superior economic benefit compared to maize planted in even rows. We recommend that this approach should be popularized and put into widespread use.

### The advantages of intercropping alfalfa with maize

Intercropping plays an important part in traditionally intensive agriculture and has captured attention for its efficient utilization of limited resources [21], [34]. Among numerous agricultural intercropping modes, legume/cereal intercropping has been most successful, with a long history and several apparent advantages [12], [35]–[36]. Common patterns include intercropping peanuts [37], soybeans [38] or cowpeas [39] with maize. However, few studies have investigated the potential advantages of intercropping alfalfa with maize [24], [40]–[41], and there has been no systematic study attempting to identify whether it can provide sustained high yield and economic incomes while taking investments and environmental factors into consideration or analyze the constraints limiting its popularization. Furthermore, previous intercropping studies have planted their maize in even rows [23], [28]. To our knowledge, no field data are available for intercropping alfalfa with maize in alternating wide and narrow rows.

Owing to differences in the traits, growth and development characteristics of alfalfa and maize (Figure S4), the alfalfa/maize intercropping system resulted in temporal and spatial complementarity, which optimized resource utilization and promoted intercropping advantages [27]. The details are presented as follows:

First, as a perennial forb, alfalfa turns green in early spring, grows fast and mainly covers the soil by late April, whereas maize is sown in early or mid-May. We found that when alfalfa was entering the first flowering stage, maize was still a seedling with canopy lower than that of alfalfa. Thus alfalfa and maize occupy complementary spatial and temporal niches, resulting in complementarity in light interception (Figure 2) and facilitating the circulation and diffusion of air (especially CO_2_) in the composite population. This result is consistent with many other studies [42]–[43]. The increase of light transmission of intercropped alfalfa produces an edge effect and enhances the LAI of alfalfa (Figure 5), thereby significantly improving the hay yield of alfalfa in the first cut, especially when using the IMA1 strategy (Figure S2). Furthermore, the hay yield of alfalfa in the first cut accounted for more than 50% of the total hay yield [19]; therefore, the increase in hay yield in the first cut made a great contribution to total hay yield (Figure S2).

Second, between alfalfa first cut and third flowering stage, maize achieves a period of peak growth, became taller than alfalfa; occupying a more advantageous position in the intercropping system so as to make full use of light, heat and other resources. This effect is particularly evident immediately after the second cutting of alfalfa, when intermediate and bottom light transmission of intercropped maize is dramatically enhanced (Figure 2B and C); producing better growing conditions for intercropped maize and accelerating dry matter accumulation (Figure S3). As a result, intercropped maize has an opportunity for recovery [44], partly compensating for the reduction in the maximum dry matter and grain yield caused by competition with alfalfa. In addition, many studies have demonstrated that there is nitrogen transfer from legumes to cereals in intercropping systems [45]–[46]. Especially, alfalfa, a perennial leguminous forb, has a strong ability to fix nitrogen, and its fixed and transferred nitrogen contributed as much as 30% to the total N accumulated in the associated grass [47]–[48]. Therefore, we speculate that there would be nitrogen transfer from alfalfa to maize in the alfalfa/maize intercropping system, which can enhance soil nitrogen availability, improve soil physical and chemical properties and be responsible for the improved growth and development of intercropped maize. It should be noted that the relatively favorable precipitation and allocation in the growing season in 2011 and 2013 was also beneficial in alleviating the growth inhibition of intercropped maize caused by water deficit (Figure 1). Consequently, the combined effects of these factors narrowed the grain yield gap between intercropped maize and monoculture maize and promoted the formation of intercropping advantages.

Third, alfalfa can continue to grow for around one month after harvesting of maize (Figure S4), providing enough time for alfalfa roots to store adequate carbohydrates to overwinter [23]. Thus, the temporal and spatial differentiation in alfalfa/maize intercropping system avoided wasting light, heat, water, air and other natural resources, prolonged photosynthetic effective time, increased photosynthetic effective area, and ultimately enhanced group yield (Table 2).

Although intercropping alfalfa with maize occupies largely complementary aboveground ecological niches, there is belowground competition. Alfalfa has deep strong roots, that can penetrate>10 m into the soil but mainly proliferate at a soil depth of 0–60 cm. The roots of maize are shallow and mostly distributed at a soil depth of 0–40 cm [49]. Despite some differentiation in root distribution, alfalfa and maize will compete in the shallow soil layers where most water and nutrients are distributed. Moreover, alfalfa has a higher evaporation coefficient and requires more water than maize [18], [50]. Therefore, when water is limited, intercropped alfalfa competes with maize for water in the shallow layers to meet its growth and development besides utilizing deep groundwater [28], which contributed to the promotion of LAI and hay yield of intercropped alfalfa at the flowering stage (Figure 5 and S1; Table 2).

However, the strong competition of alfalfa with maize significantly reduced SWC of the intercropping system, especially at the first flowering stage of alfalfa and between its second and third flowering stage when maize was in the period of seedling and peak growth with high water demand (Figure 3). The water stress restrained the growth and development of intercropped maize, particularly in the early growth stage (first 80 days post-emergence) (Figure S3), and significantly declined the LAI and grain yield at harvest time (Figure 4; Table 2).

The competitiveness for resources of intercropped species differs due to competition and complementary in the intercropping system. Generally, cereals are considered to have a competitive advantage over legumes and have a decisive influence on the total yield in annual legume/cereal intercropping systems [31], [51]–[52]. However, Zhang et al. [23] studied an alfalfa/maize intercropping system and found that alfalfa had much greater competitiveness than maize, and that alfalfa yield dramatically influenced the total yield of the intercropping system. Our results are consistent with this study. In our two intercropping modes (IMA1 and IMA2), the competitiveness for resources of alfalfa was much stronger than that of maize, and alfalfa hay yield was significantly improved whereas maize grain yield was significantly reduced. In addition, any reduction in maize yield was more than offset by increased alfalfa yield (Table 2). Thus both intercropping systems have consistently accomplished a successful tradeoff between complementarity and competition, enhanced land use capability and achieved a significant yield and output value advantage over monoculture, except that in 2011 the two intercropping patterns achieved a similar yield and OVPUA compared to MMW (Table 2).

When investment is considered, both IMA1 and IMA2 can improve economic benefits in a great extent relative to MMW in both years. This is because alfalfa is a perennial legume that does not require ploughing, sowing and fertilization after the establishment. Compared to maize, planting alfalfa can reduce costs by at least 584 USD ha^−1^ (based on the fertilizer level and seeding rate in our study and in which fertilizer, seed and farming labor savings were 376, 113 and 95 USD ha^−1^). In this way, relative to MMW, the IMA1 mode not only increased output value (+187.77 USD ha^−1^ in 2011 and +641.57 USD ha^−1^ in 2013), but also reduced investment (−135 USD ha^−1^), thus enhanced economic benefits by 322.77 USD ha^−1^ and 776.57 USD ha^−1^ in 2011 and 2013, respectively. Similarly, the final economic benefit of IMA2 was also enhanced by 106.79 USD ha^−1^ in 2011 and 804.96 USD ha^−1^ in 2013, as compared to MMW.

The higher total yield and output value of alfalfa/maize intercropping in 2013 than 2011 (Table 2) could be attributed to the following two aspects. First, the favorable higher amount and better distribution of precipitation in 2013, especially in the growing season, improved the SWC (Figure 3). Thus water competition between alfalfa and maize was minimized, nutrient availability to crops was enhanced [53] and crops obtained a superior condition for growth and development. Meanwhile, the improved growth conditions made alfalfa reach the flowering stages earlier, especially for the second and third times. Thus the co-growth time of alfalfa and maize with strong competition was shortened, and maize obtained a more unconstrained condition to utilize resource and grow. Second, with the intercropping year's increase, alfalfa roots descend into deeper soil layers [49] and could extract water at greater depths when the upper soil horizons get drier, which would improve the growth conditions of intercropped maize; at the same time, it has been proved that alfalfa can fix and transfer more nitrogen to the associated grass as time goes on [54], thus, it is likely that alfalfa could transfer more nitrogen to intercropped maize and improve its growth and development.

Therefore, the comprehensive benefit of alfalfa intercropped with maize was not completely stable and had a certain variation with the changes of rainfall and planting years. It should be noted that the economic benefit of intercropping is also strongly correlated with local market prices of alfalfa and maize [28]. In addition, environmental stress (e.g. pest, disease and the freezing injury to alfalfa in early spring) should also be taken into account to evaluate the valorization of the comprehensive benefits of the intercropping system, and it could severely reduce crops productivity and the economic returns. However, compared to monoculture, intercropping enhanced crops resistance to stress and reduced the management costs and economic losses per unit area [14]–[16]. Overall, both of the two intercropping strategies have potential to improve economic incomes and are superior to monoculture. This result is in accordance with many other studies and manifests the advantages of intercropping [16], [28], [55]. Moreover, the intercropping mode of IMA1 was superior to that of IMA2, regardless of land use efficiency, total yield or output value.

Nevertheless, the maize/alfalfa intercropping system still remains relatively unpopular for a number of reasons. First, because of the distinct cultural requirements of each crop, it is difficult to manage the two crops together with existing farm machinery. In this study, all crop management was performed using manual labor. Sowing, fertilizer application, weed control and harvest were conducted separately due to growth and management differences between the two crops. It was also required to manage pest and disease control independently for each crop - when either crop suffered from pests or disease, appropriate pesticides and safeguards were selected to minimize influence on the other crop. It is clear that managing the intercropping system is more complicated and inconvenient than management of a monoculture system. In order to simplify management of this intercropping system, it will be necessary to integrate a multidisciplinary body of knowledge to develop efficient machinery and cautiously advocate the popularization of this intercropping system on a moderate scale to realize yield and economic advantages [56]. Secondly, although the 383,000 km^2^ FPA and NEC areas [1] are well suited for the popularization of this intercropping system, local farmers have traditionally utilized a single cropping system and are likely unaware of intercropping systems, especially an intercropping system mixing a food crop and forage legume. Under current conditions, these farmers are more likely to select traditional planting strategies with simple and convenient management schedules and are little concerned about sustainable food production, animal husbandry or eco-environmental security (local investigation and personal communications). Therefore, the dissemination of efficient intercropping technologies and expert technical guidance, as well as financial support of government will necessarily play an important role in putting alfalfa/maize intercropping into practice in the FPA of NEC [56]. Finally, further research is required to assess the long-term benefits of the composite crop population and its responses to rainfall, planting years and environmental stresses (pest, disease and freezing injury), in order to avoid agronomic risks and economic loss.

## Conclusions

Based on the above analyses, we conclude that alfalfa/maize intercropping has obvious advantages in grain yield and economic incomes; it guarantees regional food security and provides superior forage. Therefore, this intercropping strategy can help maximize use of limited land resources and promote sustainable development of agriculture and animal husbandry. Moreover, nitrogen fixation and transfer from alfalfa to maize can improve soil fertility and reduce fertilizer investment [19]. Furthermore, alfalfa hay yield increases continuously throughout the first five years [18], providing sustainable economic benefits.

With rising demand for meat, egg, milk and nutrient balance, China is giving increasing importance to the development of animal husbandry and investing the forage industry [9]. Therefore, alfalfa market prices are likely to increase. In this way, planting alfalfa will not only meet the demands of the animal husbandry industry, but also promote the rapid development of local economies. In addition, the multiple-year coverage of alfalfa on the soil can alleviate wind erosion and water erosion, improving the environment of planting areas [19]. In conclusion, there are clear and significant economic, social and ecological benefits in alfalfa/maize intercropping, and maize intercropped with one row of alfalfa was identified as the optimal strategy.

## Supporting Information

Figure S1 **Daily variation dynamics of air temperature from February to April in 2012.** T-highest  =  highest temperature in a day, T-lowest  =  lowest temperature in a day.
(TIF)

Figure S2 **Alfalfa hay yield at three different flowering stages under monoculture and intercropping.** MA  =  alfalfa monoculture, IMA1  =  maize intercropped with one row of alfalfa in the wide rows, IMA2  =  maize intercropped with two rows of alfalfa in the wide rows. Different lower case letters for the same flowering stage in one year indicate significant difference, and significant differences of alfalfa total hay yield for one year between different cropping patterns are indicated by different capital letters (*P* <0.05). Values  =  means ± SE.
(TIF)

Figure S3 **Accumulation dynamics of maize aboveground dry matter under monoculture and intercropping.** MME  =  monoculture maize in even rows, MMW  =  monoculture maize in alternating wide and narrow rows. The other symbols are the same as for Figure S2.
(TIF)

Figure S4 **Growth dynamics of alfalfa and maize in the intercropping system.**
(TIF)
