# Supplementary figures and images for: Alfalfa (Medicago sativa L.)/Maize (Zea mays L.) Intercropping Provides a Feasible Way to Improve Yield and Economic Incomes in Farming and Pastoral Areas of Northeast China
Source: PLoS One. 2014 Oct 16;9(10):e110556. doi: 10.1371/journal.pone.0110556 (PMC4199727; doi:10.1371/journal.pone.0110556)

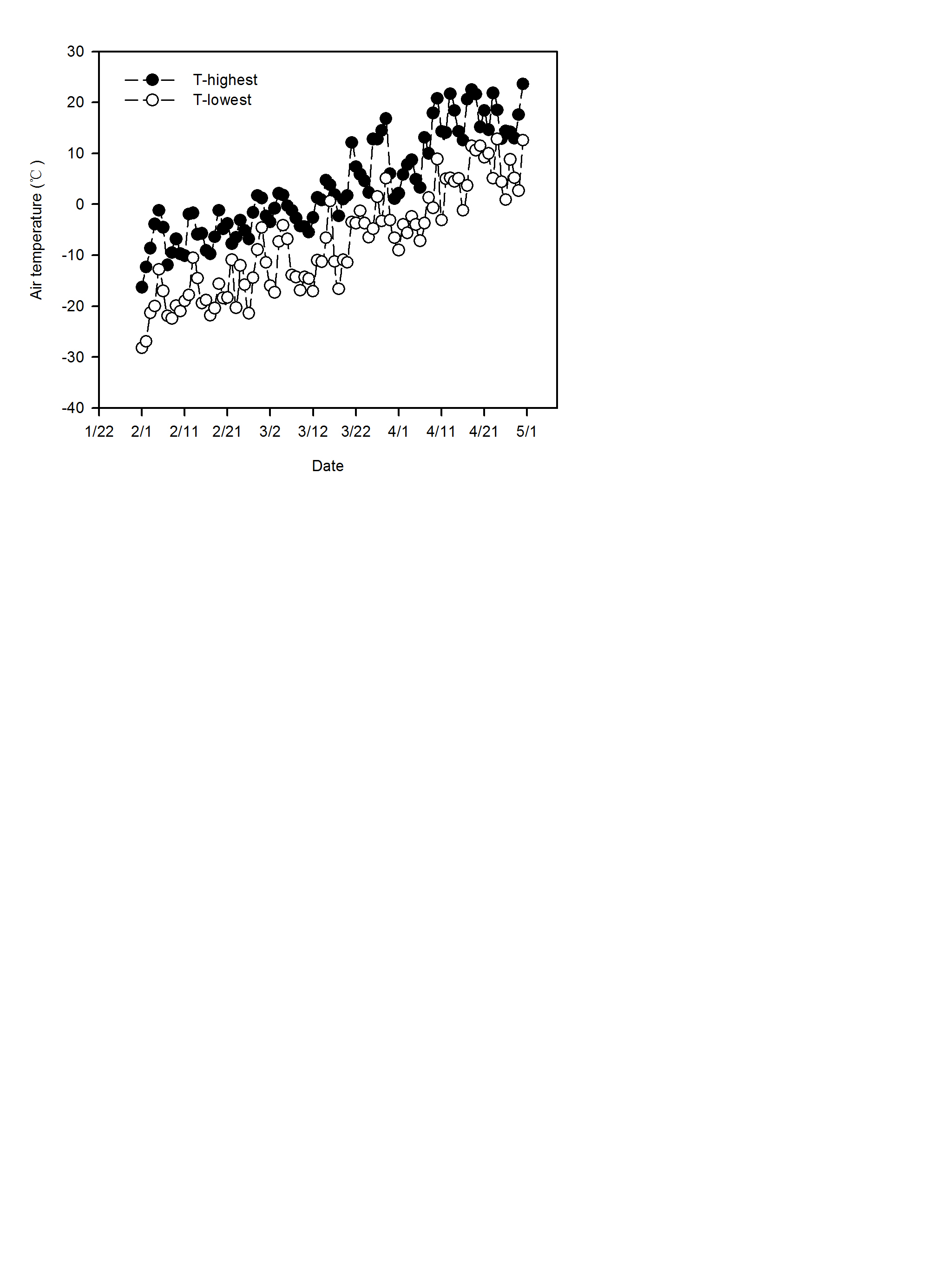

Supplement: Figure S1 — Daily variation dynamics of air temperature from February to April in 2012. T-highest = highest temperature in a day, T-lowest = lowest temperature in a day. (TIF) [file pone.0110556.s001.tif]

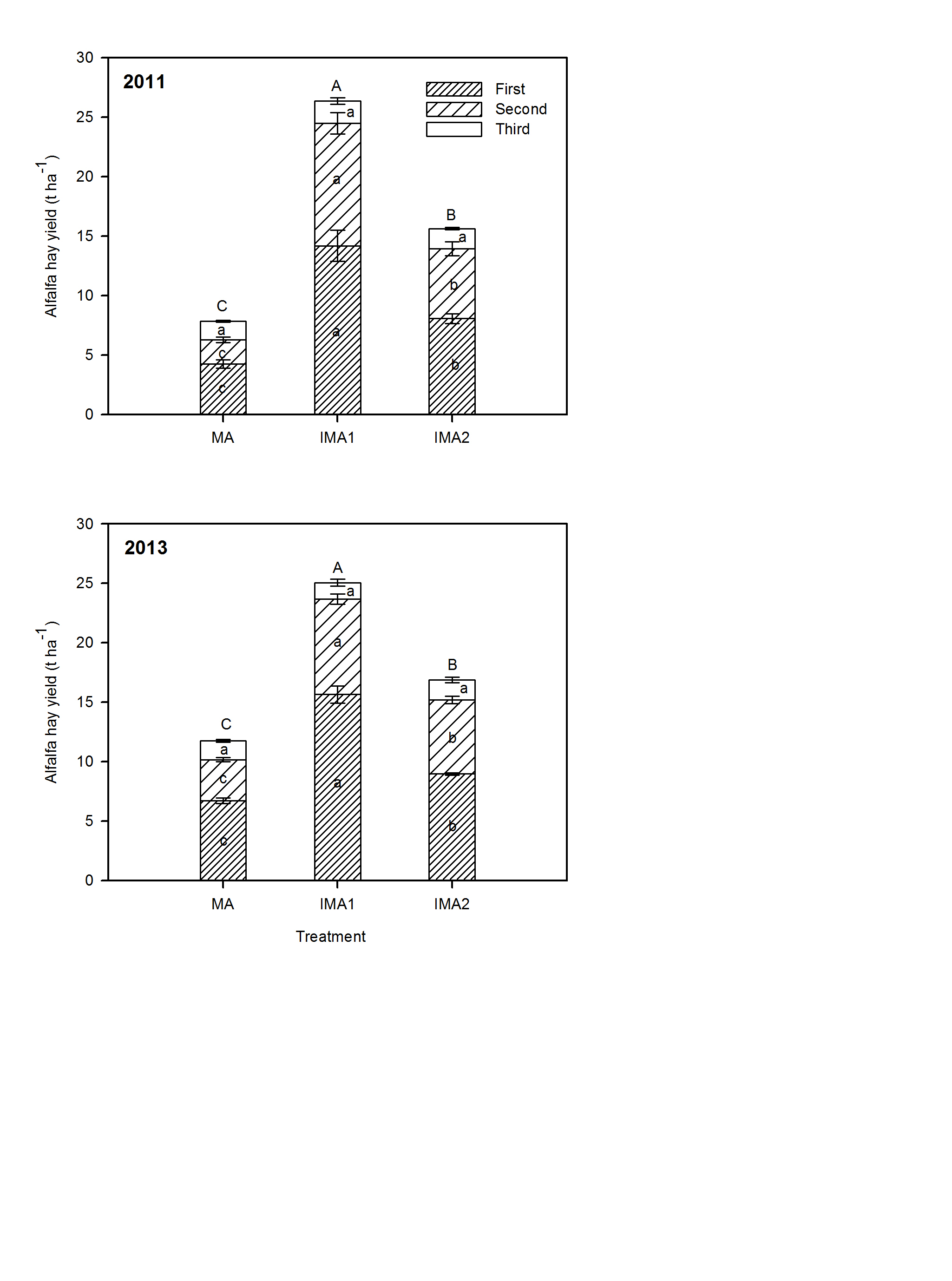

Supplement: Figure S2 — Alfalfa hay yield at three different flowering stages under monoculture and intercropping. MA = alfalfa monoculture, IMA1 = maize intercropped with one row of alfalfa in the wide rows, IMA2 = maize intercropped with two rows of alfalfa in the wide rows. Different lower case letters for the same flowering stage in one year indicate significant difference, and significant differences of alfalfa total hay yield for one year between different cropping patterns are indicated by different capital letters (P <0.05). Values = means ± SE. (TIF) [file pone.0110556.s002.tif]

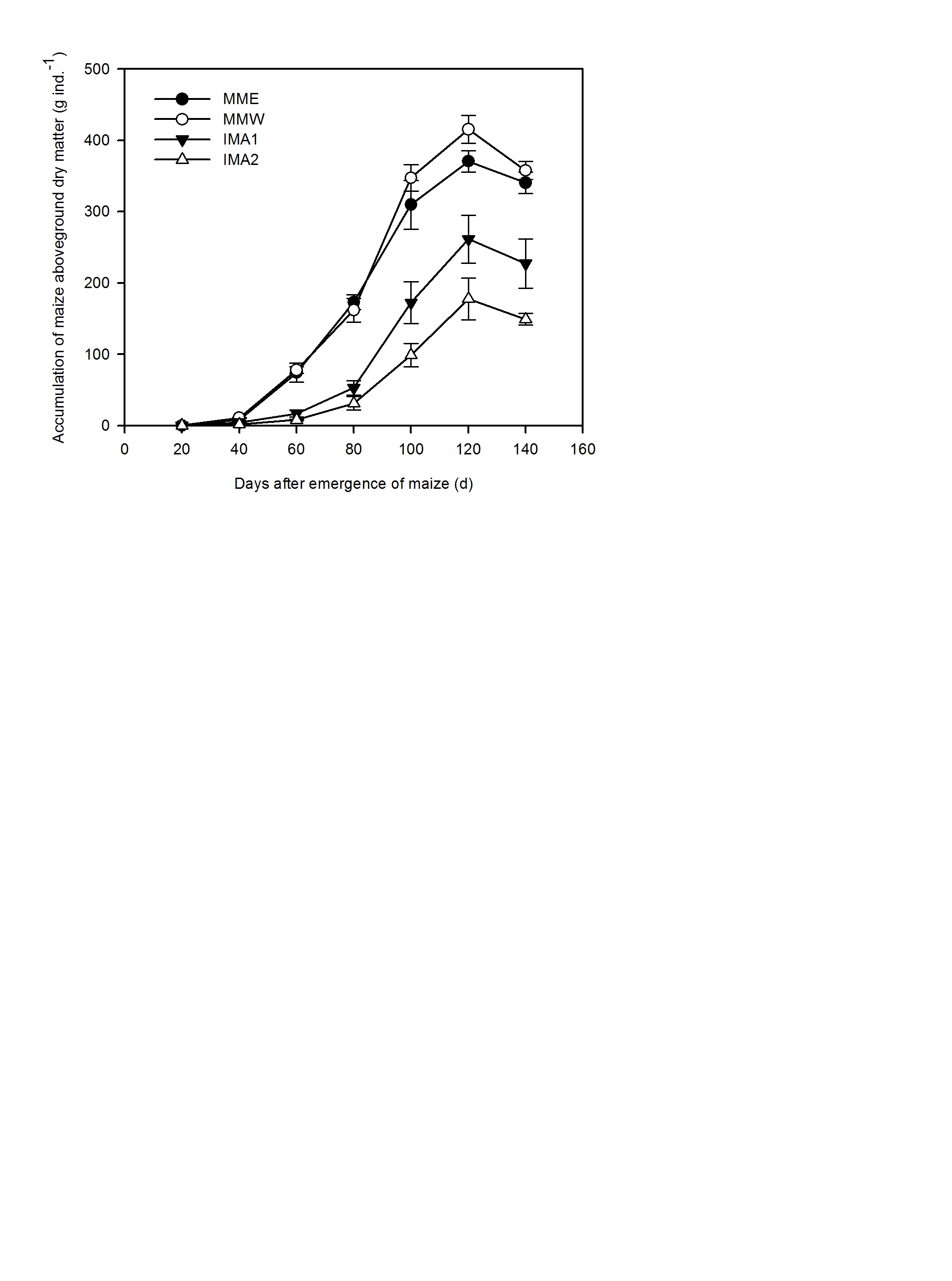

Supplement: Figure S3 — Accumulation dynamics of maize aboveground dry matter under monoculture and intercropping. MME = monoculture maize in even rows, MMW = monoculture maize in alternating wide and narrow rows. The other symbols are the same as for Figure S2. (TIF) [file pone.0110556.s003.tif]

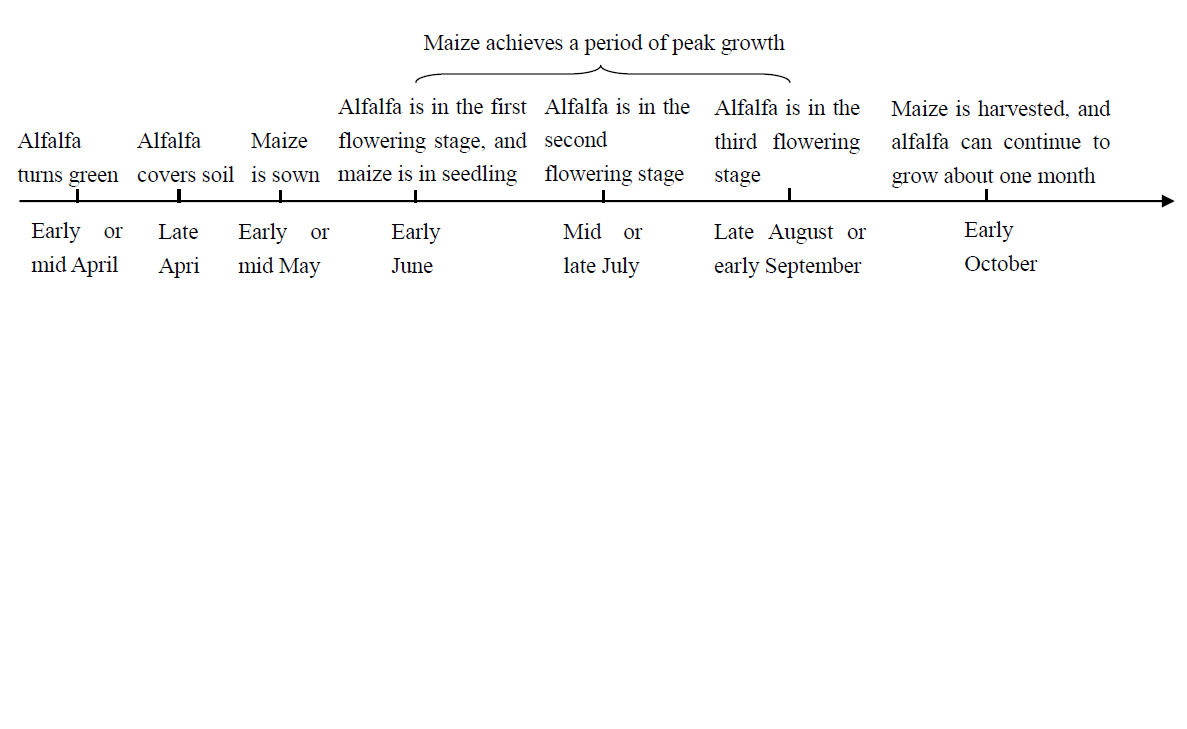

Supplement: Figure S4 — Growth dynamics of alfalfa and maize in the intercropping system. (TIF) [file pone.0110556.s004.tif]
